# Commentary: The Code for Facial Identity in the Primate Brain

**DOI:** 10.3389/fnhum.2017.00550

**Published:** 2017-11-14

**Authors:** Bruno Rossion, Jessica Taubert

**Affiliations:** ^1^Face Categorization Lab, Psychological Sciences Research Institute and Institute of Neuroscience, Université Catholique de Louvain, Louvain-la-Neuve, Belgium; ^2^Service de Neurologie, Centre Hospitalier Universitaire de Nancy, Nancy, France; ^3^School of Psychology, University of Sydney, Sydney, NSW, Australia

**Keywords:** monkey brain, individual face recognition, human specificity, ventral temporal cortex, decoding

A recent study analyzed the variation in firing rates among neurons of the monkey brain in response to a large set of humanoid face images (Chang and Tsao, 2017). The paper has received considerable attention, especially in the popular press, because it claims to have “cracked the code” underlying individual face recognition in primates. Here, we put this study in its historical scientific context and explain why this claim is not substantiated.

Variations in the firing rate of face-selective neurons to images of different individual faces has long been reported in the monkey infero-temporal (IT) cortex, with population coding proposed as a mechanism for the recognition of individual faces (Baylis et al., 1985; Rolls, 1992). Hence, the alternative account that a single neuron fires for a single face (a “grandmother cell”) is a straw man, which has never been seriously considered by this scientific community. Furthermore, an earlier study already showed that physically similar faces were represented by similar patterns of discharges across neurons sampled in monkey IT, with coding along two axes explaining most of the variance in the response pattern (Young and Yamane, 1992). The lack of reference to these previous studies might be because, at that time, functional magnetic resonance imaging in monkeys was not available to guide single cell recordings. Yet, specifically targeting face-selective patches, does not prevent the study of Chang and Tsao (2017) suffering from other methodological shortcomings. For instance, the study relies on a high-dimensional (*n* = 50) psychometric face identity space, testing neurons with monotonic response profiles, but at no point establishes a null hypothesis; i.e., what conditions or stimulus manipulations would result in poor decoding/encoding performance? If another linear space was created via the same process but with round fruits as the input images rather than faces, would we expect a different pattern of responses from face-selective neurons? Is a different pattern even possible when using such a high-dimensional space? Why would face identity decoding benefit from pooling neurons' responses across two monkey brains?

There are, however, more fundamental issues. As in previous studies cited above, these findings are thought of as revealing face identity coding in humans. Yet, there is no evidence that, like humans, monkeys rely primarily on facial information to individualize conspecifics in their natural environment, let alone are experts at individualizing human faces. In laboratory settings, monkeys need extensive training (i.e., hundreds of trials) to reach above chance performance at discriminating individual conspecific faces (Parr et al., 2000; Pokorny and de Waal, 2009). In these studies, even after conditioning, significant drops of performance are observed with novel images, indicating a reliance on image-based cues. Monkeys' viewing preference for novel vs. habituated images of conspecifics might also be entirely due to low-level image attributes and/or non-identity related cues (e.g., eye gaze direction) (Pascalis and Bachevalier, 1998). The argument that monkeys (also other nonhuman primates; Martin-Malivel et al., 2006) rely on image-based information is supported by the lack of a performance cost for inverted faces—arguably the clearest marker of expert face recognition in humans (Rossion, 2008)—when monkeys individualize conspecific faces (Rosenfeld and Van Hoesen, 1979; Bruce, 1982). Note that ambiguity regarding the inversion cost in monkeys could be attributed to confusion of inversion with rotation effects (Parr et al., 2000), or comparing trained upright to untrained inverted stimuli (Dahl et al., 2013). Other similar studies also measuring responses to transformed face stimuli do not support the view that monkeys rely on qualitatively similar processes as humans when individualizing faces (Parr et al., 2000, 2012).

If monkeys lack human-like expertise at individual face recognition, why would variations in single neuron spike rates in their brain successfully decode human faces? This issue concerns both the limitations of anatomico-functional brain homologies across species, and the interpretation of a classifier performance. In humans, the cortical face network is primarily located in the ventral occipito-temporal cortex (VOTC; Rossion et al., 2012). However, the temporal lobe in monkeys is much smaller and relatively thinner than in humans even after considering relative body size (Rilling and Seligman, 2002), their VOTC lacks a (right lateralized) fusiform gyrus critical for individual face recognition in humans (Meadows, 1974), and does not include face-selective regions. Rather, monkeys' cortical face network, where face-selective neurons are typically recorded, is in the superior temporal sulcus (STS), in addition to a small region in the lateral anterior temporal lobe. Strikingly, complete bilateral STS lesion in monkeys only has a small unselective effect on individual face recognition (Heywood and Cowey, 1992). However, it causes a severe deficit in discriminating eye gaze direction, a biologically relevant cue in monkeys, coded in synchrony with head orientation in their STS (Perrett et al., 1985). Interestingly, humans also have STS face-selective regions involved in coding dynamic aspects of faces such as deviations in eye gaze direction rather than face identity (Puce and Perrett, 2003). Hence, the STS face-selective network might be partly homologous across these two primate species sharing a common ancestor 25–30 million year ago. In contrast, a right lateralized VOTC face-selective network may have evolved selectively in the human lineage in response to an increase in morphologic and genetic interindividual variability in the face (Sheehan and Nachman, 2014) and social pressure to rapidly discriminate among many individuals.

In summary, the recently reported study (Chang and Tsao, 2017) does not constitute a step forward in our understanding of the neural mechanisms of individual face recognition in the human species. Instead, it serves as a timely reminder that successful pattern decoding of stimuli in a psychometric space with a linear classifier might be observed even when the units sampled are not important for the presumed function. To conclude with an analogy, although different complex sounds, such as different spoken words, would likely elicit different patterns of activity across neurons in the monkey auditory cortex, could high decoding performance and subsequent reconstruction of the words be used to understand language comprehension mechanisms in humans?

## Author contributions

All authors listed have made a substantial, direct and intellectual contribution to the work, and approved it for publication.

### Conflict of interest statement

The authors declare that the research was conducted in the absence of any commercial or financial relationships that could be construed as a potential conflict of interest.
